# Classification of Food Additives Using UV Spectroscopy and One-Dimensional Convolutional Neural Network

**DOI:** 10.3390/s23177517

**Published:** 2023-08-30

**Authors:** Ioana-Adriana Potărniche, Codruța Saroși, Romulus Mircea Terebeș, Lorant Szolga, Ramona Gălătuș

**Affiliations:** 1 Basis of Electronics Department, Faculty of Electronics, Telecommunication and Information Technology, Technical University of Cluj-Napoca, 400114 Cluj-Napoca, Romania; lorant.szolga@bel.utcluj.ro (L.S.); ramona.galatus@bel.utcluj.ro (R.G.); 2 Department of Polymer Composites, Institute of Chemistry “Raluca Ripan”, Babes-Bolyai University, 400294 Cluj-Napoca, Romania; liana.sarosi@ubbcluj.ro; 3 Communications Department, Faculty of Electronics, Telecommunication and Information Technology, Technical University of Cluj-Napoca, 400114 Cluj-Napoca, Romania; romulus.terebes@com.utcluj.ro

**Keywords:** UV spectrum, deep learning, neural network, spectroscopy

## Abstract

Food additives are utilized in countless food products available for sale. They enhance or obtain a specific flavor, extend the storage time, or obtain a desired texture. This paper presents an automatic classification system for five food additives based on their absorbance in the ultraviolet domain. Solutions with different concentrations were created by dissolving a measured additive mass into distilled water. The analyzed samples were either simple (one additive solution) or mixed (two additive solutions). The substances presented absorbance peaks between 190 nm and 360 nm. Each substance presents a certain number of absorbance peaks at specific wavelengths (e.g., acesulfame potassium presents an absorbance peak at 226 nm, whereas the peak associated with potassium sorbate is at 254 nm). Therefore, each additive has a distinctive spectrum that can be used for classification. The sample classification was performed using deep learning techniques. The samples were associated with numerical labels and divided into three datasets (training, validation, and testing). The best classification results were obtained using CNN (convolutional neural network) models. The classification of the 404 spectra with a CNN model with three convolutional layers obtained a mean testing accuracy of 92.38% ± 1.48%, whereas the mean validation accuracy was 93.43% ± 2.01%.

## 1. Introduction

Food additive use has increased since the twenty-first-century lifestyle demands a quicker pace for everyday activities [1,2]. Humans do not have as much time as their ancestors for cropping vegetables, breeding animals, and preparing meals. Their usual routine revolves around work, home duties, and leisure activities. Although people do not grow their crops, they still need food for survival. 

Food is nowadays easily accessible [3]. Basic ingredients and packed products can be bought from local markets and supermarkets, whereas fresh cooked products are available in restaurants, pastries, and bakeries. Most products can also be ordered through delivery applications [4,5].

The downside of buying cooked/packed food products is not knowing the ingredients and their quality. Also, products may contain one or more food additives [6]. Food additives are extremely important for the food industry. These substances counteract spoilage, prolong the storage duration, or give the product a distinguished flavor, color, or texture [7]. The use of food additives has increased the variety of food products available for sale [7], has allowed consumers to taste local food products from distant countries [8], and has allowed companies to produce large quantities of food without the risk of food spoilage within the production-delivery period. Food additives are considered safe for consumption considering the acceptable daily intake (ADI). The ADI is the estimated quantity of a certain additive that a person can consume daily without any health risks [9].

Six categories of food additives were identified based on their functionality: preservatives, coloring agents (natural or synthetic colorants), flavoring agents (sweeteners, flavor enhancers), texturizing agents (emulsifiers, stabilizers), nutritional agents (vitamins), and other additives (catalysts) [7]. Aspartame, acesulfame potassium, and saccharin are some commonly used sweeteners, whereas sodium benzoate and potassium sorbate are preservatives [10]. Preservatives fight bacteria within food and prevent the product from spoilage for longer periods than normal, whereas sweeteners provide a sweet taste and fewer calories (or none) than other sugar types [7].

Aspartame is a non-saccharide artificial sweetener that is rapidly metabolized when consumed. The digestive enzymes transform it into methanol, phenylalanine, and aspartic acid [11]. It is commonly used to provide a sweet taste to beverages and food products. The ADI established by the FDA (US Food and Drug Administration) is 50 mg/kg of body weight (bw) per day, whereas according to the EFSA (European Food Safety Authority), the recommended ADI is 40 mg/kg bw/day [12]. Acesulfame potassium (AceK) is a chemically synthesized sweetener considered safe for human consumption. The ADI recommended by the JECFA (Evaluations of the Joint FAO/WHO Expert Committee on Food Additives) is 0–15 mg/kg bw/day. It is commonly used for confectionary products, including sweets and candies.

Saccharin, another artificial sweetener, is also used in sweets (cookies, jams) and soda drinks. It is a white crystalline powder, 300–400 times sweeter than table sugar, with an ADI of 0–5 mg/kg bw, according to JECFA. Two of the most exhaustively used preservatives are sodium benzoate and potassium sorbate. These additives fight against yeast and molds within pickled vegetables, fruit products, cheese, and soft drinks [13,14]. The ADI recommended by JECFA is 0–25 mg/kg bw for potassium sorbate and 0–5 mg/kg bw for sodium benzoate.

Even though these substances are considered harmless, multiple studies have been performed on sweeteners [15,16,17,18], preservatives [19,20], and other food additives [21] to identify the effects of additives on health. Notwithstanding aspartame being considered safe for consumption, its effects on health are still very much debated. Studies have shown that aspartame consumption may impair the growth of the placenta in impregnated mice [12], affect the extravillous trophoblasts of pregnant rodents [22], and influence fertility for women of reproductive age [23]. Similarly, research studies on mice indicate that AceK might increase the risk of atherosclerosis [24], and saccharine consumption during metformin therapy could hinder the effect of the anti-diabetes medication [25]. Reported results have also illustrated that preservatives may have inhibitory effects at the molecular level [14]. Another study identified consumers’ perceptions and knowledge of food additives [26].

Various methods have been identified for the determination of food additives. Some techniques can detect only a certain additive [27,28], whereas others can simultaneously determine multiple substances [29,30,31]. The artificial sweetener, aspartame, has been detected using surface-enhanced Raman spectroscopy [27]. The analyzed samples were prepared by dissolving aspartame in mineral water samples (flavored and unflavored). The results highlight that the technique can detect aspartame within 15 s, even for samples containing other additives (flavored water samples).

Electrochemical sensors have also been employed for additive detection. These sensors were based on a molecularly imprinted polymer and could determine acesulfame potassium (AceK) concentrations between 0.1 and 17 μm in real samples [28].

Capillary electrophoresis is one technique that allows the separation and quantification of different artificial sweeteners. The technique has been used to concomitantly determine four artificial sweeteners within dietetic soft drinks and tabletop sweetener samples. It provides rapid and accurate results and does not require elaborate sample preparation, making it appropriate for food quality applications [29].

Simultaneous detection of food additives was also performed using liquid chromatography/tandem mass chromatography. This method was used to identify ten sweeteners in alcoholic/non-alcoholic beverages and food products [30]. Colorimetry was used for the quantification of sodium benzoate in juice, wine, and vinegar samples for low limits of detection of 2 μM [32], whereas terahertz spectroscopy was combined with SVM (support vector machine) for the identification of six food additives based on coumarin [33]. The proposed methods obtained are reliable, accurate, and more efficient than other detection methods. 

Over the past few years, analysis methods have been combined with machine/deep learning techniques for determining food additives or other substances within food products. Sodium benzoate and potassium sorbate were concurrently ascertained using ultraviolet spectrophotometry, the backpropagation neural network algorithm, and partial least squares regression (PLS) [34]. Sugar content in wine grapes was assessed using VNIR-SWIR (visible and near-infrared and short-wave infrared) spectroscopy and machine learning algorithms such as SVM, SVR (support vector regression), CNN (convolutional neural network), and other techniques [35]. In contrast, the determination of aflatoxin B_1_ in maize was accomplished using NIR (near-infrared) spectroscopy, Markov transition field (MTF) image coding, and a CNN [36]. Infrared spectroscopy and CNN were also used for the classification of Italian pasta [37], melamine and cyanuric acid detection [38], yali pear inspection [39], and polysaccharide detection [40]. 

Considering the large number of studies that analyze the effects of food additives [15,16,17,18,19,20,21] and the various methods that are used for the additives’ identification [27,28,29,30,31], it can be stated that the identification, classification, and analysis of food additives are important, up-to-date subjects of research. 

This work uses artificial neural networks (ANNs) and CNN algorithms to identify and classify food additives based on the spectra recorded in the ultraviolet region of the electromagnetic spectrum. Ultraviolet spectroscopy is combined with neural network algorithms to identify five food additives: two preservatives (sodium benzoate and potassium sorbate) and three artificial sweeteners (aspartame, acesulfame potassium, and saccharin). The objective was to analyze and categorize the spectrum of one-additive solutions and two-additive solutions using coupling spectroscopy with neural network algorithms. 

## 2. Materials and Methods

This study aimed to classify five additives based on their ultraviolet (UV) spectrum. According to the Beer–Bouguer–Lambert Law, the absorbance of a liquid sample depends on the molar concentration (molarity) of the solution, the optical path, and the molar absorptivity. Since the molar concentration influences absorbance, spectra with different absorbance values can be acquired for each additive, constructing a varied and large database. 

Liquid samples were prepared by dissolving the additives into distilled water. Two types of samples were prepared. The first category included samples that contained only one food additive dissolved in distilled water (simple solutions). The second category consisted of samples created by mixing two food additives previously dissolved in distilled water (mixed solutions or two-additive solutions). Table A1 presents the five food additives that were selected for this study (three sweeteners and two preservatives). 

### 2.1. UV-Visible Spectrophotometer

The liquid samples were analyzed using the Jasco V-750 UV-Visible Spectrophotometer (Hachioji, Tokyo, Japan). This device has a wavelength range between 190 and 900 nm, with a wavelength accuracy of ±0.2 nm at 656.1 nm. The spectral bandwidth is 1 nm, with photometric accuracies of ±0.0015 Abs (for 0 to 0.5 Abs) and ±0.0025 Abs (for 0.5 to 1 Abs) [41]. 

Before the spectrum acquisition, the baseline was recorded using distilled water in both the sample and reference cuvettes. The first measurements were performed using simple solutions. To initiate the spectrum acquisition, 3 cm^3^ of the sample was deposited in the sample cuvette using a pipette. The cuvette was cleaned after each solution using distilled water.

### 2.2. Simple Solutions

The simple solutions were prepared by diluting each additive in 5 cm^3^ of distilled water. Table A2 presents the calculated and measured mass of solute necessary for obtaining five solutions. Lower concentrations required small quantities of additives, which could not be measured using the laboratory scale (the device could measure quantities down to 0.0001 g). Therefore, concentrated solutions were diluted. The molar concentrations were halved by mixing 1.5 cm^3^ of the concentrated sample with 1.5 cm^3^ of distilled water. Table 1 presents the maximum and the minimum molar concentration and the number of samples that were analyzed for each additive. A total of 193 spectra were acquired for one-additive solutions. 

### 2.3. Two-Additives Solutions

The two-additive solutions were obtained by combining two samples of similar or different concentrations. For each sample, the mass of the substance was measured and then the substance was dissolved in 5 cm^3^ of distilled water. The mixture was prepared by adding 2 cm3 of the first sample and 2 cm^3^ of the second. Since both samples contained distilled water when the samples were combined, the molar concentrations of each substance were halved when the samples were combined. Ten different mixtures (Table 2) were obtained with the five additives.

### 2.4. Deep Learning Techniques

The classification of the additives was performed using deep learning techniques. Deep learning methods process large amounts of data, identifying patterns within the dataset to provide precise predictions. One of the most famous deep learning algorithms is CNN (convolutional neural network). This algorithm automatically determines the input dataset’s important features without external supervision. CNN has been successfully used for texture feature detection [42], hyperspectral image classification [43], medical diagnosis [44], and facial recognition [45] and has also been associated with optical techniques for food analysis [37,38,39]. The most popular CNN architectures are AlexNet, GoogLeNet, ResNet, X-ception, Inception-V4, and others [46]. 

The structure of a neural network was developed based on the human brain and the nervous system. A CNN is constructed using several convolutional layers followed by sub-sampling layers (pooling layers), a flatten layer, and multiple fully connected layers [39]. The convolutional layer is built of convolutional filters known as kernels. The kernel size defines the filter mask’s dimensions applied to the input dataset. For a 1D-CNN, the kernel size could be set to 3, 5, or larger values, whereas for a 2D-CNN, the kernel size would have to be two-dimensional (e.g., 3 × 3). 

The convolutional layers create feature maps that are sub-sampled by the pooling layer. In other words, the pooling layers save the most significant features within pooled featured maps. The methods that could be used in a pooling layer are max pooling, average pooling, global average pooling, gate pooling, global max pooling, and others [39]. In some cases, these layers decrease the general performance of the network. A pooling layer saves the most important features but focuses on determining the correct location of the feature, resulting in relevant information loss [46]. 

The data within the pooled feature maps must be delivered to the fully connected layers, but firstly, it needs to pass through a flattening process, transforming the pooled featured maps into a vector [46]. The flatten layer is connected to fully connected layers. These layers are positioned at the end of the network, with all the neurons from one layer being connected to all the neurons from the preceding layer. The fully connected layers are also the main parts of an ANN (artificial neural network). Therefore, a CNN could be described as an ANN with one or more convolutional and pooling layers.

ANNs present an input layer and one or more hidden and output layers. Each layer presents neurons that are defined by weights and a bias. Data are passed from one layer to another depending on the output of each neuron. Neurons are activated based on non-linear activation functions such as Sigmoid, Tanh (hyperbolic tangent), ReLU (Rectified Linear Unit), Leaky ReLU, or NoisyReLU [46]. The most utilized activation function is ReLU due to its low computational load. This function converts the input data into positive numbers [46].

The classification is performed at the end of the fully connected layer. The output layer employs loss functions to establish the error between real and predicted outputs. The real outputs are also called labels, whereas the predicted outputs are known as predictions. Loss functions that can be utilized include cross-entropy (Softmax), Euclidean, Mean Square Error (MSE), Hinge, and others. The Softmax loss function is used for assessing the performances of a model, the Euclidean loss function is used for regression applications, and the Hinge loss function is utilized for binary classification [46]. 

The loss functions can be minimized using optimizers. These methods are mathematical functions that enhance the performance of the network. Adam (Adaptive Moment Estimation) is the most commonly used optimizer due to its low computational load and memory efficiency [46]. Other optimizers include Momentum, Mini-Batch Gradient Descent, Stochastic Gradient Descent, and Batch Gradient Descent [46]. Other parameters influencing the network’s performances are batch size and the number of epochs. The batch size defines a subset of samples the network uses before the model parameters are updated. The number of epochs can be defined as the number of iterations for which the model would be trained. 

A CNN needs to learn the dataset’s features to assess new data and make predictions. Therefore, the input dataset is split into three datasets, each used for a different purpose. The training dataset helps the network learn the most significant features and usually contains 80% of the dataset. The rest of the data are used to calculate the accuracy of the network by comparing the predictions with the actual classes. The results obtained after testing are represented as confusion matrixes. A third dataset (the unseen data) can be used for validation to eliminate the possibility of data overfitting.

A CNN model can obtain good training and testing results, but when faced with new unseen data, it might be unable to correctly classify the data. Overfitting might appear due to many correlated parameters [46], the training dataset size may be too reduced or may contain irrelevant information. The opposite effect might also appear and is known as underfitting. In this case, the model is not trained enough, so it cannot associate the input data with the correct output. 

**Figure 1:**
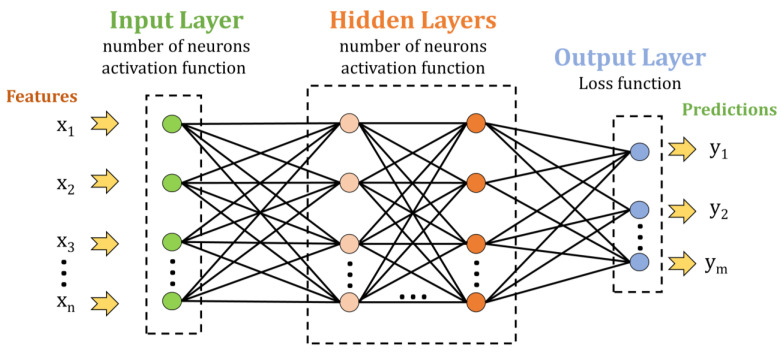
General architecture of ANN model with an input layer, multiple hidden layers, and an output layer.

This study tested several ANN and CNN architectures to establish which would be more appropriate for classifying UV spectra. The architecture selection was performed using trial-and-error until the best performance was achieved. Sequential models for ANN and CNN were implemented in the Python programming language [47]. The initial classification was performed using an ANN since their sequential model only contains an input layer, hidden layers, and an output layer, as is presented in Figure 1. 

The validation accuracy was tested for a network with one and two hidden layers. The input layer was a fully connected layer with 128 neurons that used an ReLU activation function. The hidden layers were also fully connected layers that contained 256 neurons and that used ReLU as an activation function. The number of neurons in the output layer varied because the network was tested initially for one-additive solutions (the output layer contained 5 neurons), for two-additive solutions (10 neurons), and for all the samples (15 neurons). 

Convolutional and pooling layers were added to the ANN model. The CNN was built with one, two, and three convolutional layers, followed by pooling layers to test how the accuracy changes with the increase in convolutional layers. The performances of the model were also tested without pooling layers.

**Figure 2:**
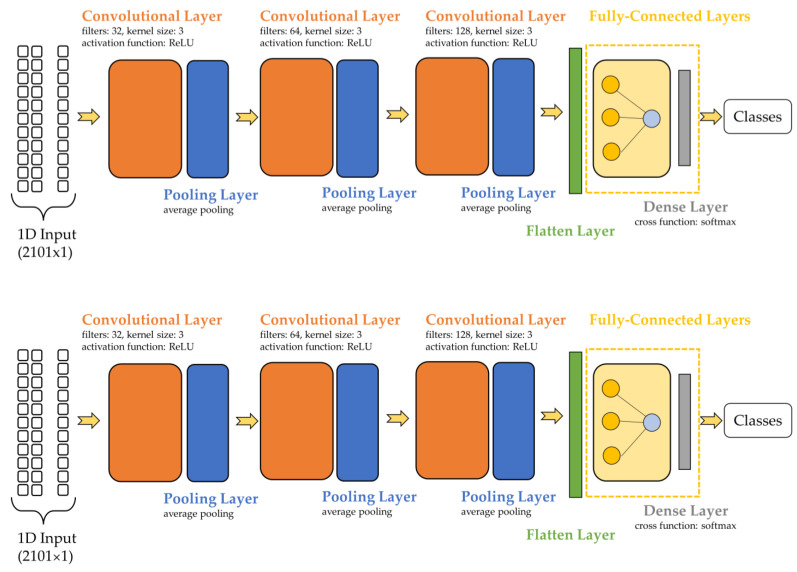
CNN with three convolutional layers followed by pooling layers.

The sequential model for the CNN is presented in Figure 2. The first convolutional layer presented 32 filters, a kernel size of 3, and an ReLU activation function. The second and third layers were similar, differing only in the number of filters (second layer 64, third layer 128). The methods used for the pooling layer included average pooling for the 1-dimensional dataset. A flatten layer was positioned between the convolutional and fully connected layers. The input layer was built using 128 neurons and an ReLU activation function. The hidden layers of the ANN used the ReLU activation function and had 256 neurons per layer. The cross-function utilized in the output layer was Softmax. 

## 3. Results

### 3.1. UV Spectra of Simple Solutions

**Figure 3:**
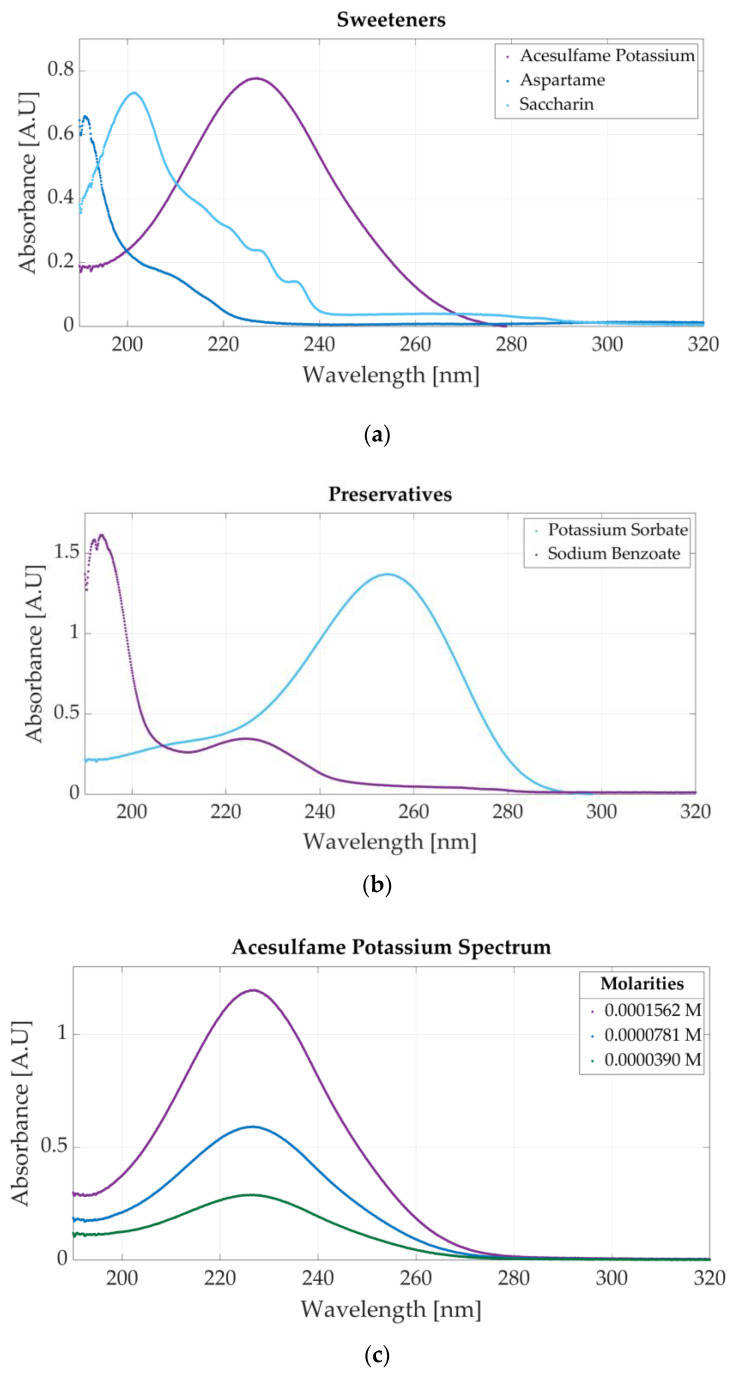
Ultraviolet spectra of food additives: (**a**) UV spectrum of acesulfame potassium, aspartame, and saccharin; (**b**) UV spectrum of sodium benzoate and potassium sorbate; (**c**) spectra for three molar concentrations of acesulfame potassium obtained through dilutions.

The spectrometric measurements performed using the Jasco V-750 UV-Visible Spectrophotometer (Hachioji, Tokyo, Japan) show that each additive has a specific spectrum, making their classification possible. The spectrum of the sweeteners is presented in Figure 3a. The molar concentrations of the samples are 0.0000625 M acesulfame potassium, 0.00000976 M aspartame, and 0.0000117 M saccharin. Acesulfame potassium presents an absorbance peak at 226 nm, aspartame at 191 nm, and saccharin at 201 nm.

**Figure A1:**
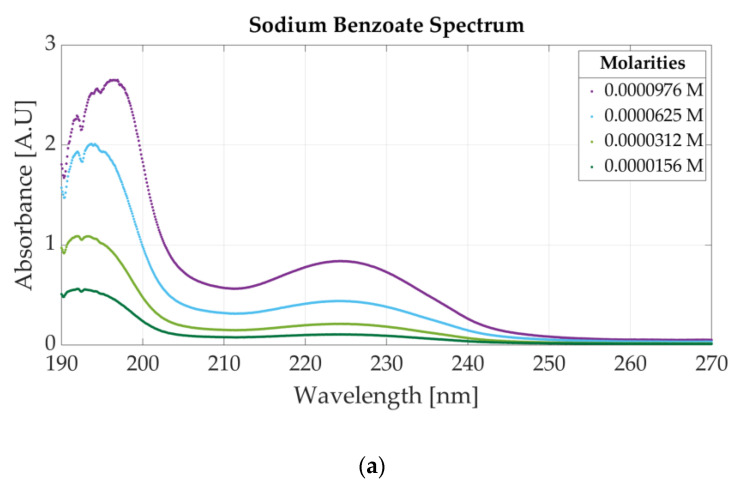

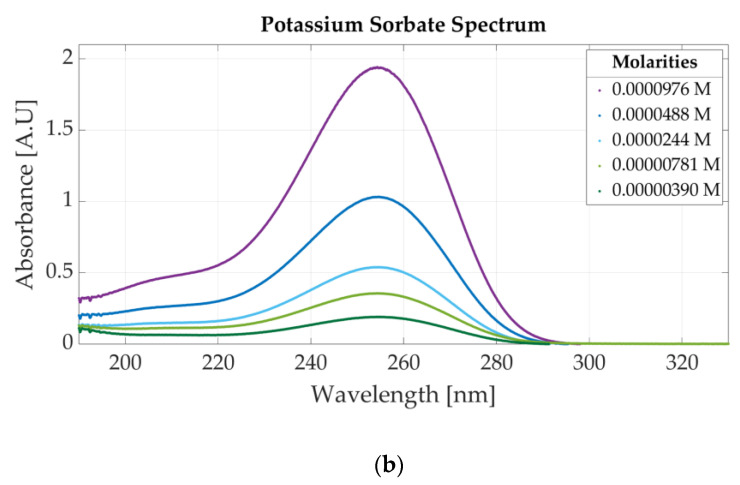
Ultraviolet spectra of food additives: (**a**) UV spectrum for four concentrations of sodium benzoate: (**b**) UV spectrum for five concentrations of potassium sorbate.

Figure 3b presents the ultraviolet spectrum for a sample of 0.0000312 M potassium sorbate and 0.0000468 M sodium benzoate. The absorbance peak specific to potassium sorbate appears at 254 nm, whereas sodium benzoate has two peaks located at 193 nm and 224 nm. The spectra of four solutions of acesulfame potassium are represented in Figure 3c. The three solutions are obtained by diluting a sample of 0.0001562 M acesulfame potassium. For these solutions, the only change that appears is related to the value of the absorbance peak given by the Beer–Bouguer–Lambert Law. The change in absorbance due to a molar concentration change can also be observed in Figure A1a for four samples of sodium benzoate and Figure A1b for five samples of potassium sorbate.

**Figure 4:**
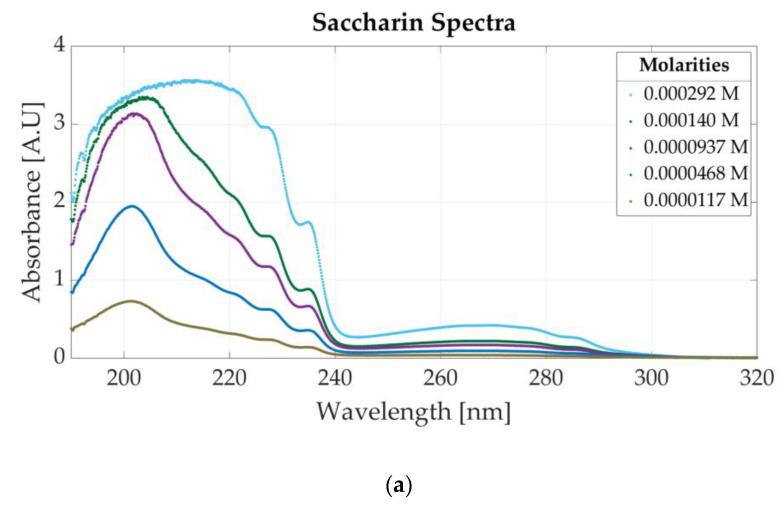

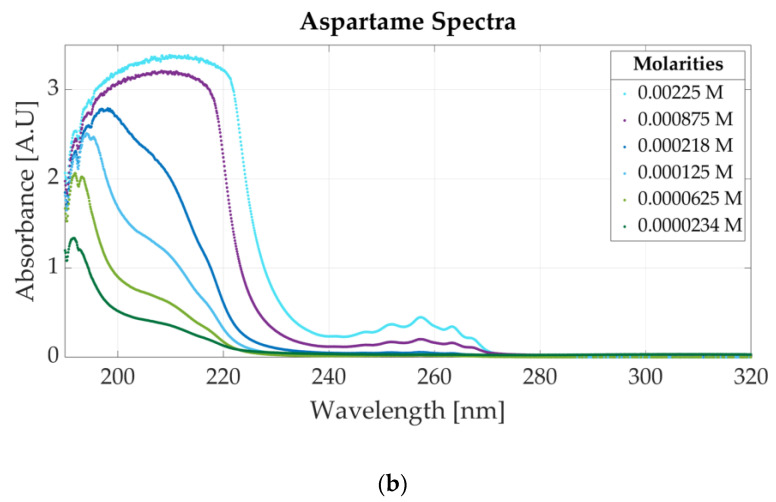
Ultraviolet spectra of food additives: (**a**) UV spectra for different concentrations of saccharin; (**b**) UV spectrum for different concentrations of aspartame.

Figure 4a presents the spectrum of six saccharin samples with different molar concentrations. The molar concentrations of the samples vary between 0.000292 M and 0.0000117 M. For lower concentrations than 0.0001 M, the absorbance peak located at 201 nm is visible. For higher concentrations of saccharin (e.g., 0.000292 M), high absorbance values appear in the left part of the spectrum (190 to 240 nm), which might indicate the saturation of the photodetector. Based on the results, the dynamic absorbance range is concluded to vary between 0 and 3 absorbance units (A.U). For absorbance values higher than 3 A.U, the photodetector can no longer accurately measure the absorbance values within 190 nm and 240 nm. Therefore, for concentrations higher than 0.0000937 nm, the absorbance peak associated with saccharine is at 269 nm.

Similarly, Figure 4b represents the spectrum of six samples of aspartame with molar concentrations between 0.00225 M and 0.0000234 M. For concentrations higher than 0.0002 M, five peaks appear within 240 nm and 270 nm (the highest being located at 257 nm), whereas on the right side of the spectrum, high absorbance values are measured (the 191 nm peak is no longer visible). The five peaks are no longer visible for a concentration of aspartame lower than 0.0002 M, but a peak appears between 190 and 200 nm.

### 3.2. UV Spectra of Two-Additive Solutions

**Figure 5:**
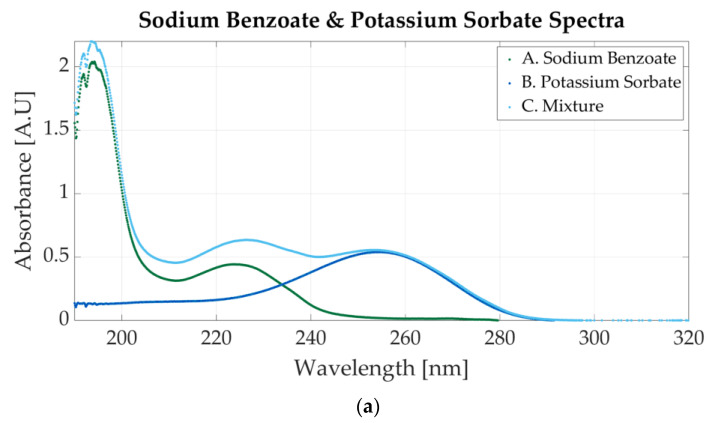

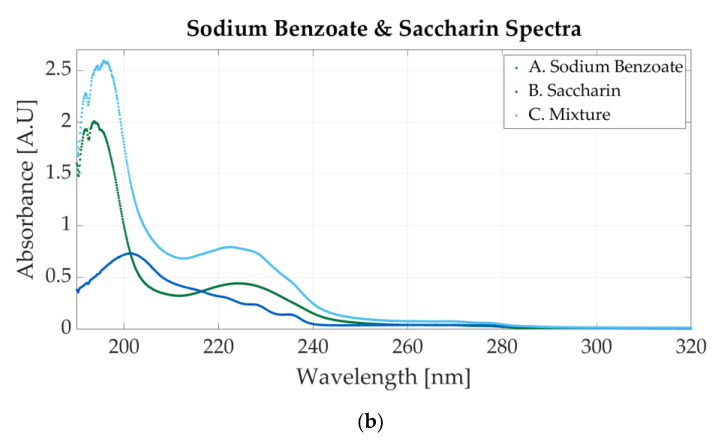
Ultraviolet spectra of food additives. (**a**) A. Sodium benzoate, 0.0000427 M; B. potassium sorbate, 0.0000244 M; C. 0.0000488 M sodium benzoate and 0.0000244 M potassium sorbate; (**b**) A. sodium benzoate, 0.0000546 M; B. saccharin, 0.0000117 M; C. 0.000058 M sodium benzoate and 0.0000117 M potassium sorbate.

Figure 5a shows the spectrum of a mixture of sodium benzoate and potassium sorbate and the spectra of two simple solutions (one for sodium benzoate and one for potassium sorbate). The mixture contains 0.0000488 M sodium benzoate and 0.0000244 M potassium sorbate. The one-additive solutions have similar molar concentrations to those of the mixture (0.0000427 M for sodium benzoate and 0.0000244 for potassium sorbate).

The spectra of one-additive samples do not match the spectra of the mixture, because the concentrations of sodium benzoate are different (0.0000488 M in the mixture and 0.0000427 M in the simple solution). The one-additive spectra represented are not the spectra of the two solutions that were mixed. When two samples are combined, the molar concentration of each sample is halved (if using equal parts of each solution) due to the distilled water in the samples. Therefore, the spectra of the one-additive solutions, before the mixture, would have had higher absorbance peaks (compared to the mixture).

Figure 5a shows that the absorbance values for simple solutions and for the mixtures have similar values, which indicates that they are not the spectra of the two pure samples that were mixed. The one-additive solutions are presented to highlight that the mixture spectra contain the absorbance peaks representative of each additive. Potassium sorbate has an absorbance peak at 254 nm, whereas sodium benzoate presents two peaks at 224 nm and 193 nm, as presented in Figure 3b. The three peaks are also visible in the mixture’s representation.

Sodium benzoate is also mixed with saccharin. The spectrum is represented in Figure 5b. The molar concentrations for the one-additive solutions are 0.0000546 M sodium benzoate and 0.0000117 M saccharin. The mixture contains 0.000058 M sodium benzoate and 0.0000117 M. In the mixture’s spectrum, two peaks are visible. Those peaks are specific to sodium benzoate, but the peak around 224 also presents light waves within 220 nm and 240 nm that appear in the saccharin samples’ spectrum.

**Figure 6:**
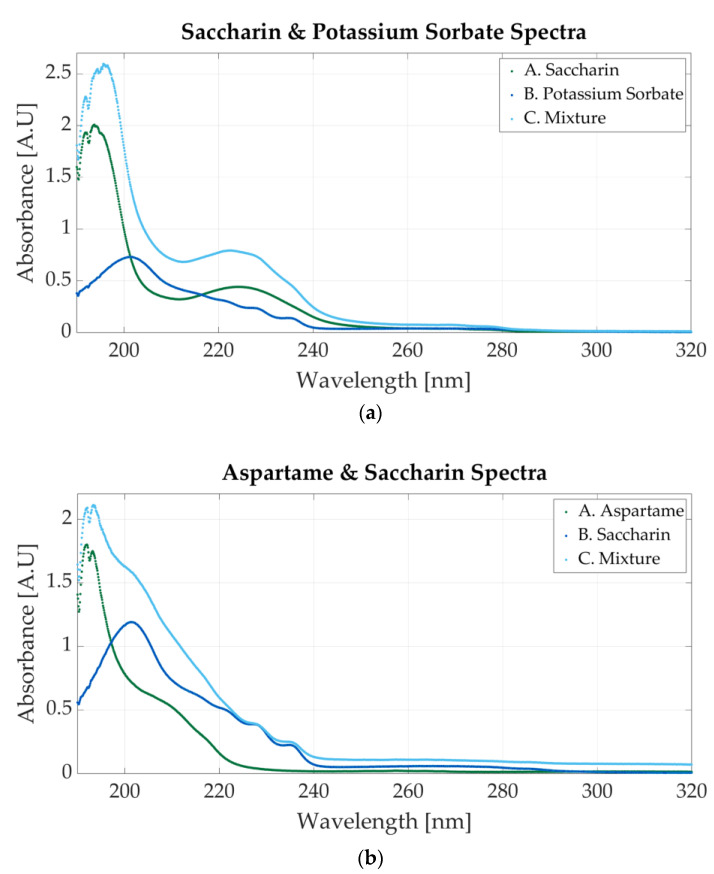
Ultraviolet spectrum of food additives. (**a**) A. Saccharin, 0.0000546 M; B. potassium sorbate, 0.0000117 M; C. 0.000058 M sodium benzoate and 0.0000127 M potassium sorbate; (**b**) A. aspartame, 0.000039 M; B. saccharin, 0.0000234 M; C. 0.000039 M aspartame and 0.0000195 M saccharin.

Figure 6a shows the spectrum of a solution with 0.0000117 M potassium sorbate and 0.0000546 saccharin. The peak located at 201 nm is representative of saccharin, whereas the one positioned at 254 nm is clear evidence of the presence of potassium sorbate. The spectra for the one-additive solutions are also represented. The molar concentration of the potassium sorbate solution is 0.0000127, whereas the molar concentration of the saccharin solution is 0.000058 M.

Figure 6b represents the first mixture containing saccharin and aspartame. The molar concentration of the samples is 0.000039 M aspartame and 0.0000195 M saccharine. The spectrum presents only one peak located around 191 nm, but a second peak, less outlined, appears at 201 nm. This peak, alongside the other two peaks within 220 nm and 240 nm, is specific to saccharin.

**Figure 7:**
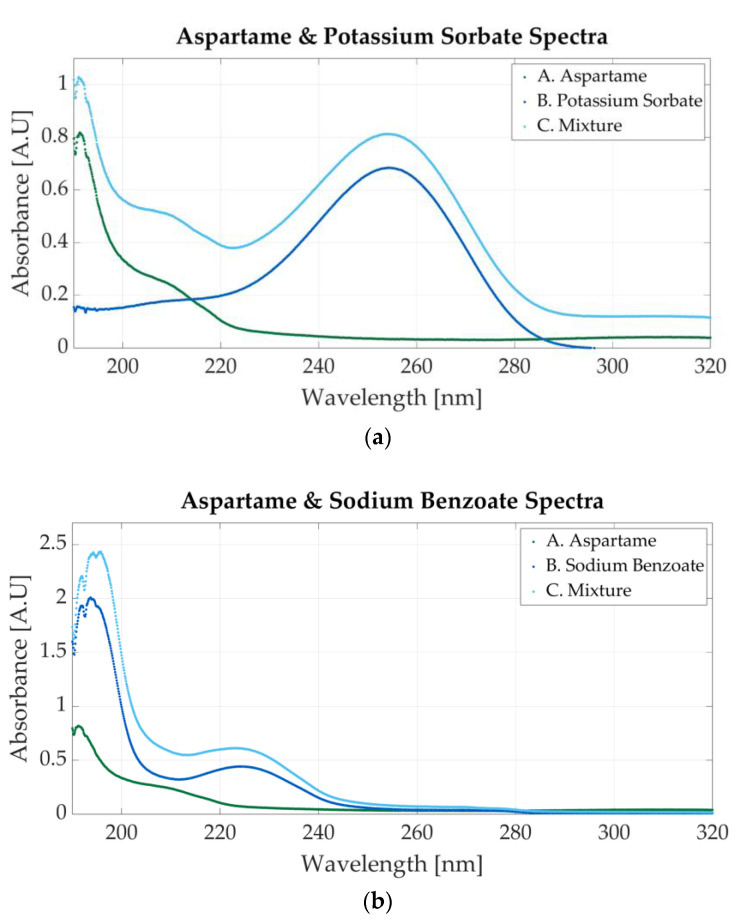
Ultraviolet spectra of food additives. (**a**) A. Aspartame, 0.0000117 M; B. potassium sorbate, 0.0000156 M; C. 0.0000915 M aspartame and 0.0000915 M potassium sorbate; (**b**) A. aspartame, 0.0000117 M; B. sodium benzoate, 0.0000546 M; C. 0.0000145 M aspartame and 0.000058 M sodium benzoate.

The spectra of two aspartame mixtures are presented in Figure 7. The first mixed solution is prepared using aspartame and potassium sorbate. The spectrum is represented in Figure 7a. The molar concentration of the sample is 0.0000915 M aspartame and 0.0000915 M potassium sorbate. The peak located at 254 nm indicates the presence of potassium sorbate, whereas the 201 nm peak is specific to aspartame. Figure 7b shows the spectrum of a mixture of aspartame and sodium benzoate. The molar concentrations of the samples are 0.0000145 M aspartame and 0.000058 M sodium benzoate. The presence of either substance might cause a peak between 190 and 200 nm since both additives present absorbance peaks in that region.

**Figure 8:**
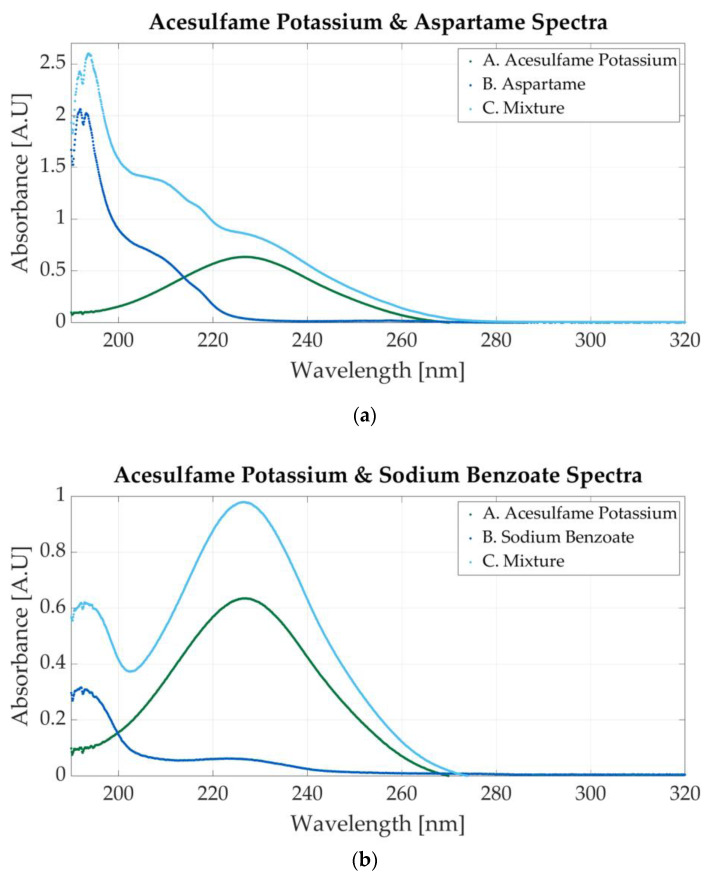
Ultraviolet spectra of food additives. (**a**) A. Acesulfame potassium, 0.00005 M; B. aspartame, 0.0000625 M; C. 0.000058 M acesulfame potassium and 0.000029 M aspartame; (**b**) A. acesulfame potassium, 0.00005 M; B. sodium benzoate, 0.00000683 M; C. 0.0000585 M acesulfame potassium and 0.00000732 M sodium benzoate.

Figure 8a illustrates the spectrum of the mixture between acesulfame potassium and aspartame. The spectrum of the mixtures presents a peak at 191 nm (specific to aspartame) and a slight peak around 226 nm peak (specific to acesulfame potassium). The molar concentration of the sample is 0.00058 M acesulfame potassium and 0.000029 M aspartame. 

The mixture in Figure 8b contains 0.0000585 M acesulfame potassium and 0.00000732 M sodium benzoate. The absorbance peaks for acesulfame and sodium benzoate are very close. In Figure 3a,b, acesulfame presents an absorbance peak at 226 nm, whereas sodium benzoate peaks at 224 nm. The peak in the mixture’s spectrum may indicate the presence of both substances. The concentration of the additives is important. The spectrum represented in Figure 8b contains a low concentration of sodium benzoate (0.00000732 M); therefore, it can be stated that the peaks located at 226 nm are caused by acesulfame. A second peak at 193 nm is surely an indicator of sodium benzoate.

**Figure 9:**
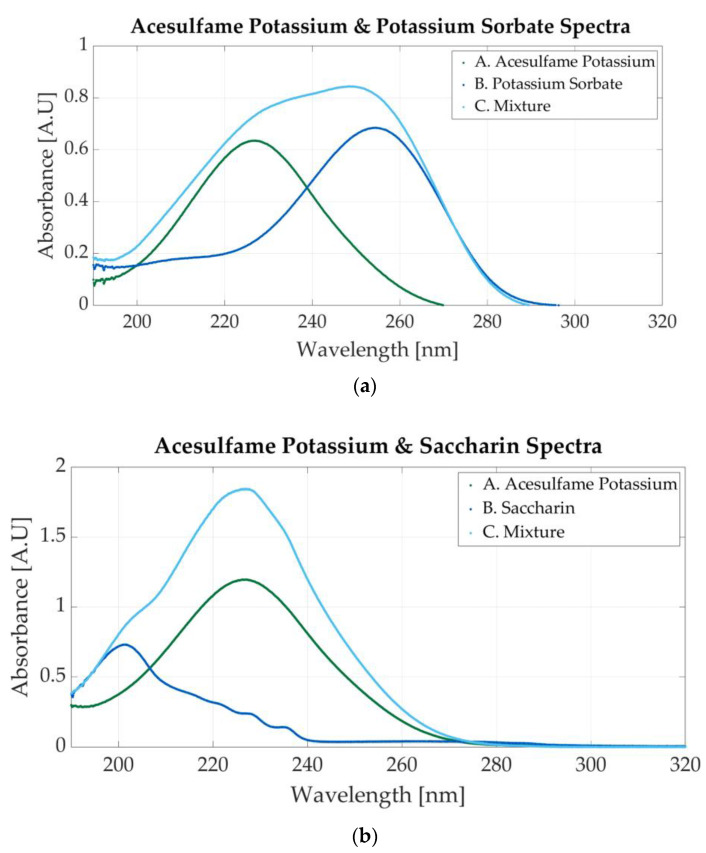
Ultraviolet spectra of food additives. (**a**) A. Acesulfame potassium, 0.00005; B. potassium sorbate, 0.0000156 M; C. 0.0000585 M acesulfame potassium and 0.0000219 M potassium sorbate; (**b**) A. acesulfame potassium, 0.000156 M; B. saccharin, 0.0000117 M; C. 0.000234 M acesulfame potassium and 0.0000145 M saccharin.

Two more mixtures can be prepared with the additives. The first is composed of acesulfame potassium and potassium sorbate and is composed of acesulfame potassium and potassium sorbate, and the second is composed of acesulfame potassium and saccharin. The spectrum of a mixture of 0.0000585 M AceK and 0.0000219 M sodium benzoate is presented in Figure 9a. The absorbance peaks of acesulfame and potassium sorbate (226 nm and 254 nm, respectively) are located very close; therefore, the peak within this region is wider. The FWHM (full-width at half-maximum) is approximately 45 nm.

Acesulfame potassium is also mixed with saccharine. The molar concentration of the sample is 0.000234 M acesulfame potassium and 0.0000145 M saccharin. The absorbance peak at 226 nm is more prominent than the 201 nm peak because the molar concentration of acesulfame potassium is higher than that of saccharin. Both peaks indicate the presence of additives within the mixture, as can be seen in Figure 9b.

### 3.3. Classification Results for One-Additive Solutions

The database for one-additive solutions contained 193 data samples (26 for acesulfame potassium, 41 for aspartame, 38 for sodium benzoate, 32 for potassium sorbate, and 56 for saccharin). The database was divided into three datasets: one for training, one for testing, and one for validating the model. The data division was accomplished according to the number of samples for each category. For each class, 20% of the data were allocated for testing, while the remaining data were divided into 80% for training and 20% for validation. Therefore, the training dataset contained 122 samples, the testing dataset contained 42 samples, and the remaining 29 were used for validation. 

The first classification was performed on one-additive solutions. A numerical label between 1 and 5 was associated with each additive (1—acesulfame potassium, 2—aspartame, 3—sodium benzoate, 4—potassium sorbate, 5—saccharin). The label was utilized for the additive classification. The classification results for the testing process are displayed in confusion matrices. In these matrices, the actual labels are represented vertically, whereas the predicted labels of the analyzed samples are represented horizontally.

**Figure 10:**
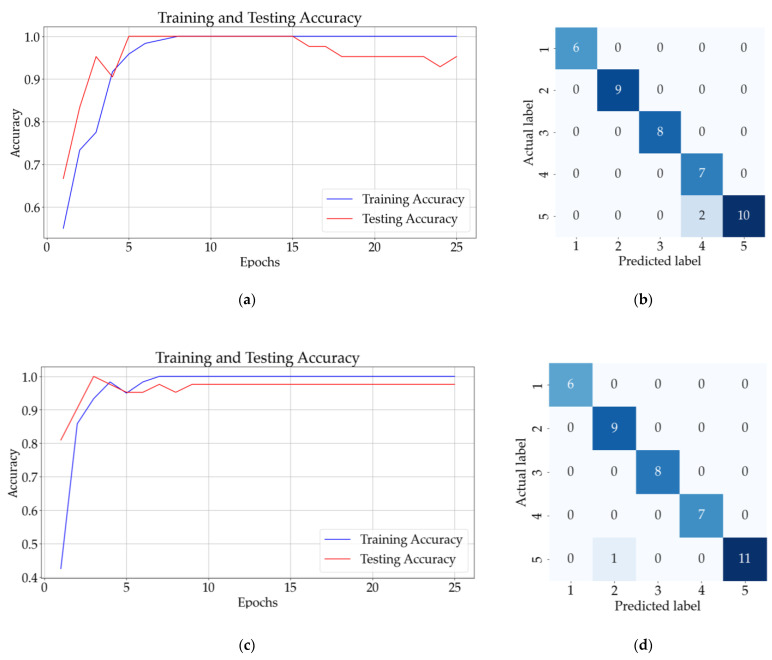
Results for one-additive solutions: (**a**) training and testing accuracy for 25 epochs for ANN with one hidden layer; (**b**) confusion matrix (ANN); (**c**) training and testing accuracy for CNN with three convolutional layers and no pooling layers; (**d**) confusion matrix (CNN).

**Figure 11:**
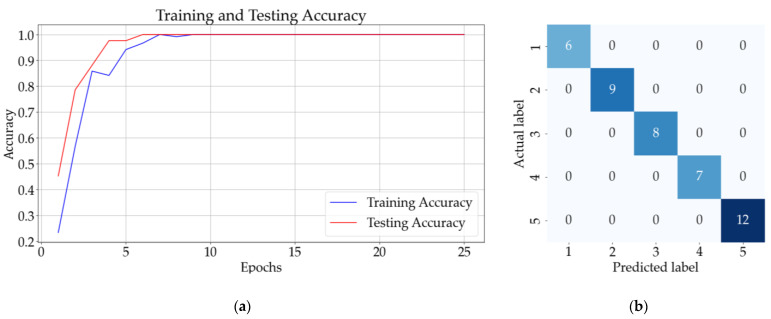
Results for one-additive solutions: (**a**) training and testing accuracy CNN with three convolutional layers followed by three pooling layers; (**b**) confusion matrix for CNN with three convolutional layers and three pooling layers.

Figure 10 presents the result for classifying the one-additive solutions using ANN and CNN. Figure 10a presents the accuracy of the ANN model (with a hidden layer) after 25 epochs, whereas Figure 10b presents the confusion matrix obtained for the testing dataset. The results for the CNN model with three hidden layers and no pooling layers are presented in Figure 10c,d, and the results for the CNN model with three hidden layers and three pooling layers are presented in Figure 11a,b.

Figure 10b shows that the ANN model incorrectly classified a sample from class 5 (saccharin) as belonging to class 2 (potassium sorbate). On the other hand, Figure 10d and Figure 11b illustrate that the CNN without the pooling layer incorrectly classified a sample from class 5 and the CNN model with pooling layers correctly identified all 42 samples.

The best mean accuracy for the testing of the model was obtained for ANN with two hidden layers. For ten consecutive classifications, the mean validation accuracy for this model was 99.67%, with a standard deviation of 1.02%. The same mean testing accuracy was obtained for a CNN with 3 convolutional layers and three pooling layers and the CNN with 3 convolutional layers and no pooling layers. The mean testing accuracy of the other models is presented in Table 3.

The classification performances of the models were tested using unseen data. The validation dataset for one-additive solutions consisted of 20% of each class of additives. The division of the database was performed randomly each time. A total of 31 samples (4 samples from class 1, 7 from class 2, 7 from class 3, 4 from class 5, and 9 from class 5) were used for the testing of the model’s classification performances.

The number of correctly classified samples was divided into the total number of samples used for testing. The mean validation accuracy was calculated for 10 successive classifications. The CNN model with one convolutional layer (no pooling layers) and the CNN with two convolutional layers followed by pooling layers were able to correctly identify 99.67% of the samples with a standard deviation of 1.02 %. The mean validation accuracy for the rest of the models is shown in Table 4.

Based on the results presented in Table 3 and Table 4, it can be concluded that the best model for the classification of the one-additive spectra was the CNN with two convolutional layers followed by pooling layers. The mean validation and testing accuracy was 99.67% ± 1.02%.

### 3.4. Classification Results for Two-Additive Solutions

**Figure 12:**
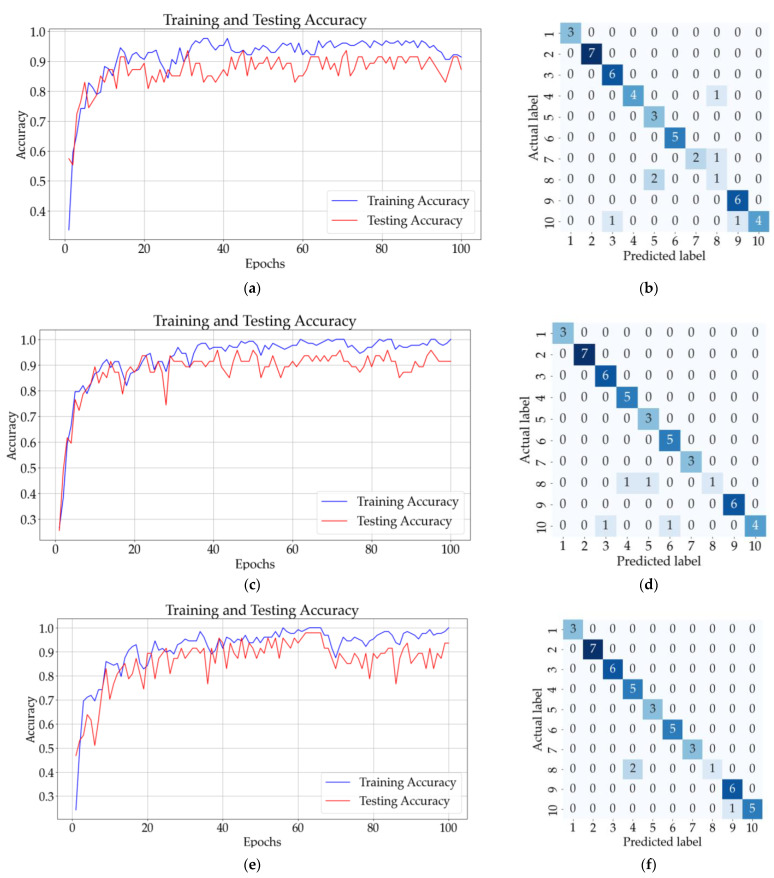
Results for two-additive solutions: (**a**) training and testing accuracy for ANN with one hidden layer; (**b**) confusion matrix (ANN); (**c**) training and testing accuracy for CNN with three convolutional layers and no pooling layers; (**d**) confusion matrix for CNN (no pooling layers); (**e**) training and testing accuracy of CNN with three convolutional layers followed by three pooling layers; (**f**) confusion matrix for CNN (with pooling layers).

The second classification was conducted on two-additive solutions. The 211 spectra have been labeled with values between 1 and 10 (the numerical labels are presented in Table 2). The classification results for ANN and CNN are presented in Figure 12. The confusion matrix from Figure 12b shows that six samples (one from class 4, one from class 7, two from class 8, and two from class 10) were incorrectly classified using ANN with one hidden layer. The testing accuracy for the ANN model, after 100 epochs, was 88%, as is presented in Figure 12a.

Figure 12c shows the confusion matrix for the CNN model with three convolutional layers and no pooling layers. Two samples from 8 were incorrectly classified as belonging to classes 4 and 5. The testing accuracy of the CNN, after 100 epochs, was 91%, as shown in Figure 12c. Figure 12f illustrates that the CNN with three convolutional layers followed by a pooling layer correctly classified all the samples apart from two samples belonging to class 8 and one sample belonging to class 10. The mean validation accuracy of the model for ten successive classifications was 92.22%, with a standard deviation of 4.3%. Table 5 presents the mean testing accuracy of the tested neural networks for two-additive solutions.

The models were tested with 36 new samples (3 samples from class 1, 5 from class 2, 4 from class 3, 4 from class 4, 2 from class 5, 4 from class 6, 2 from class 7, 2 from class 8, 5 from class 5, and 5 from class 10). Table 6 shows that the ANN models obtained validation accuracies lower than 90% with high standard deviations. The results indicate that the new samples could not be correctly identified, which was expected since the mean testing accuracy was also low and had high standard deviation (Table 5, ANN with two hidden layers, 88.88% ± 7.04%). 

On the other hand, the CNN model with one convolutional layer followed by the pooling layer obtained high validation and testing results. The mean testing accuracy was 91.94% ± 4.02% (Table 5), whereas the mean validation accuracy was 94.16% ± 3.05% (Table 6). The CNN with two convolutional layers and pooling layers was able to correctly identify 93.88% ± 4.3% of the samples but obtained only a 90.83% ± 6% (high standard deviation) testing accuracy. 

Consequently, it is considered that the best results for the classification of two-additive solutions can be obtained using a CNN model with one convolutional layer followed by a pooling layer (91.94% mean testing accuracy, 94.16% mean validation accuracy). 

### 3.5. The Classification of All Solutions

All the spectra (193 spectra for one-additive solutions and 211 spectra for two-additive solutions) were reunited to test the neural network’s accuracy using simple solutions and mixtures. One-additive solutions were labeled with values from 1 to 5, whereas two-additive solutions were labeled with values between 6 and 15 (the order of the samples was not changed). 

**Figure 13:**
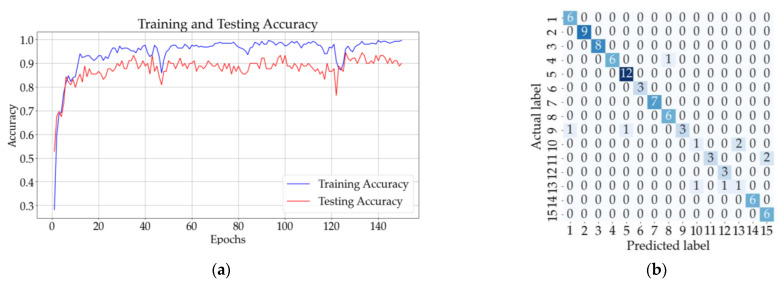

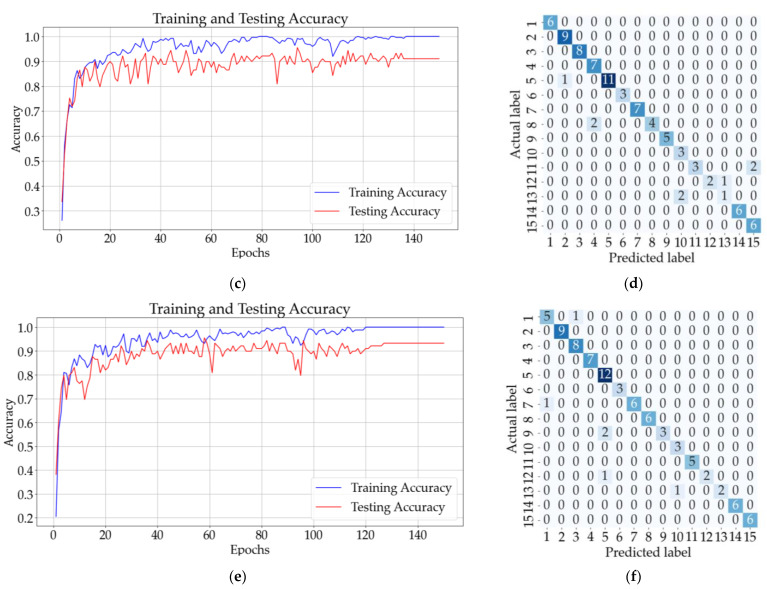
Results for all solutions: (**a**) training and testing accuracy for ANN with one hidden layer; (**b**) confusion matrix (ANN); (**c**) training and testing accuracy for CNN with three convolutional layers and no pooling layers; (**d**) confusion matrix for CNN (no pooling layers); (**e**) training and testing accuracy CNN with three convolutional layers followed by three pooling layers; (**f**) confusion matrix for CNN (with pooling layers).

Figure 13b shows that eight samples from classes 9, 10, 11, and 13 were incorrectly classified using the ANN with one hidden layer. The testing accuracy of the model was 90% after the 150 epochs. The CNN model with three convolutional layers and no pooling layers had a testing accuracy of 91%, and according to the confusion matrix illustrated in Figure 13d, only eight samples were incorrectly classified. The testing accuracy increased for the CNN with three convolutional and pooling layers (92%), but the confusion matrix from Figure 13f shows that five samples from classes 7, 9, 12, and 13 could not be classified correctly. 

The mean testing accuracy of the ANN and CNN models is presented in Table 7. The highest mean validation accuracy was obtained for the CNN model with three convolutional layers and no pooling layers (92.38% ± 1.48%). The mean validation accuracies for the other models are illustrated in Table 8.

The models were also tested using new unseen data. A total of 67 samples were used to determine which model correctly identified the most samples in ten successive classifications. The mean validation accuracy for the eight models is shown in Table 8. The best results were obtained for CNN models with 1, 2 (with and without pooling), and 3 layers. 

Considering the results obtained in Table 7 and Table 8, the highest accuracies were obtained for the CNN with three convolutional layers (92.38% ± 1.48% for testing and 93.43% ± 2.01% for validation) for the 15 classes of substances.

### 3.6. Analysis of Variance

The accuracy data presented in Table 3, Table 4, Table 5, Table 6, Table 7 and Table 8 (for testing and validation) were analyzed using a statistical method known as ANOVA (analysis of variance). ANOVA was necessary to establish if there was a significant statistical difference between the accuracy of the algorithms used for the spectra classification. The parametric one-way ANOVA assumes that the datasets are normally distributed, whereas the Kruskal–Wallis H method (known as one-way ANOVA (analysis of variance) on ranks) is a non-parametric test that can be used with nonnormally distributed data [48].

Firstly, a Shapiro–Wilk test was performed to determine the normality of the data. The Shapiro–Wilk results for one-additive solutions are presented in Table 9 (testing results and validation results). The hypothesis of normality is rejected when the *p*-value is less than or equal to 0.05. Based on the values in Table 9, the datasets were not considered to be normally distributed. 

The non-parametric ANOVA on ranks was applied on the data. The null hypothesis, that all the classes are equal, is accepted for *p*-values higher than 0.05. The analysis shows statistical differences between the accuracy of the techniques for testing (*p* = 0.02301), whereas, for validation, no difference is found (*p* = 0.29759). The results do not indicate which algorithms are more significant than the others (for testing), but it shows that the classification algorithm that is selected is relevant for obtaining higher accuracies.

The analysis was continued with a post hoc test. The Dunn test is commonly used if the Kruskal–Wallis method shows significant differences among the classes. Table 10 presents the Dunn test’s results for one-additive solutions (testing). The results (*p* = 0.42153) indicate that the ANN with two hidden layers (ANN2), the CNN with three convolutional layers (CNN3), and the CNN with two convolutional layers followed by pooling layers (CNN2*) were statistically different compared to the ANN with one hidden layer (ANN1). Statistically, the ANN2, CNN3, and CNN2* would obtain better-accuracy results for the classification of one-additive solutions. The results were compared with a significance level of 0.05 (5%).

The normality of the accuracy datasets was also tested for two-additive solutions and for all the samples. The results are presented in Table 11 and Table 12. The normality test was followed by a one-way ANOVA since the normality condition was fulfilled.

The one-way ANOVA results are shown in Table 13 (for testing) and Table 14 (for validation). The results reveal that there was a statistically significant difference in the validation accuracy datasets F(7, 27) = [2.7], *p* = 0.01526 (two-additive dataset) and F(7, 27) = [ 2.7078], *p* = 0.01503 (all samples dataset). The analysis was continued with a post hoc tests to determine which classes (algorithms) presented a significant difference. The selected post hoc test was Tukey’s HSD (Honestly Significant Difference). Based on the number of groups, the degree of freedom calculated using ANOVA (Table 14), and the significance level (0.05), the critical value of the Tukey’s HSD test was 4.41.

The results within Table 15 were compared with the critical value. The test revealed that the mean value of the accuracy was significantly different between ANN2 and CNN with two convolutional layers (CNN2) (q = 4.4252, 95%). Statistically, the CNN2 algorithm would obtain better results for the classification of the two-additive samples. 

The Tukey results for the validation dataset (all the samples) are shown in Table 16. The critical value remained the same as the one calculated for two-additive solutions (4.41). The test revealed that the mean value of the accuracy was significantly different between the CNN with one hidden layer followed by a pooling layer (CNN1*) and CNN1 and CNN2 (q = 4.7673, 95%). Statistically, the CNN1* algorithm would obtain better results for the classification of all the samples.

## 4. Discussion

This study proposes a classification method for food additives based on their absorbance in the ultraviolet domain. Spectroscopy is a non-invasive method that has been employed in various domains, such as chemistry [49], food quality control [50], veterinary diagnosis [51], wine classification [52], and water monitoring [53]. Studies [34,54] have shown that food additives exhibit strong absorbance features at wavelengths shorter than 360 nm. Therefore, UV spectroscopy can be considered a suitable approach for their classification. The use of spectroscopy alongside neural network algorithms has been reported in the literature to determine sodium benzoate and potassium sorbate in beverages [34]. 

Similar to [34], this work focuses on the classification of sodium benzoate and potassium sorbate and three additional additives that function as sweeteners (acesulfame potassium, aspartame, and saccharine). The spectra of solutions with different molar concentrations were acquired using the Jasco V-750 UV-Visible Spectrophotometer. The spectral database contained 404 spectra, 193 spectra for solutions containing only one additive and 211 for the mixture of two additives.

The classification of the samples was performed using ANN and CNN models. The performances of the models were tested for different architectures. The ANN was tested for 1 and 2 hidden layers, whereas the CNN models were built with 1, 2, or 3 convolutional layers followed or not by pooling layers.

Three classifications were performed. The first one included only simple solutions. As depicted in Table 5 and Table 6, the best model that can be used for the classification of the one-additive solutions was the CNN with two convolutional layers followed by pooling layers. The mean validation and testing accuracy was 99.67% ± 1.02%.

For the classification of two-additive spectra, the best results were obtained with the CNN model with one convolutional layer followed by a pooling layer. The model obtained high validation and testing results. The mean testing accuracy was 91.94% ± 4.02% (Table 7), whereas the mean validation accuracy was 94.16% ± 3.05% (Table 8).

The last classification was performed on the entire database. The CNN model with three convolutional layers was the best choice for the classifications of the 404 spectra, obtaining a mean testing accuracy of 92.38% ± 1.48%. The model also performed well on unseen data, obtaining a validation accuracy of 93.43% ± 2.01% for the classification of the 15 classes of substances. The overall results show that CNN obtained better results than ANN for spectra classification without signs of overfitting or underfitting. Also, the ANOVA analyses followed by post hoc tests indicate that, statistically, the best classification accuracies can be obtained using CNN algorithms. 

Future research will focus on three main aspects. The first one regards the dimensions of the spectral dataset. A total of 404 spectra were used for this study. The spectra were acquired for one-additive solutions (193 spectra) and two-additive solutions (211 spectra). For a one-additive solution, more than 25 spectra were acquired for each of the five classes, whereas for two-additive solutions, the minimum number of samples per class was 12. It is desired to enlarge the database with more spectra for two-additive solutions to include at least 25 samples per class and see the changes that appear in the spectrum when mixing solutions with other molar concentrations than the ones presented in this study. Adding supplementary spectra for two-additive solutions could improve the model’s training and validation accuracy. 

Secondly, the spectrum of new mixtures could be included in the dataset. Table 17 shows all the possible mixtures that can be obtained by mixing 3, 4, or 5 additives. The sample preparation and spectrum acquisition are time-consuming processes. The mass of the solute needs to be measured, the quantity of the solvent needs to be accurately measured to obtain the desired molar concentration, and dilutions need to be prepared to obtain various concentrations and absorbance values. Considering the average time per sample to be 7 min, it would require 5425 min (approximately 90 h) to prepare at least 25 solutions per class and acquire their spectra. 

Future research could include other food additives, such as coloring agents, texturizing agents, or nutritional agents. The analysis of samples containing five or more additives is crucial because, usually, in food products/beverages, there are more than two additives. The ingredients found within a product are important because they assist customers in apprehending what they are consuming. Identifying food additives is important since their long-term effects are still very disputed [14,22,23,24]. Also, studies have researched the influence of nutritional labels on people’s food choices [55], the importance of front-of-pack nutritional labels [56], and the awareness of consumers regarding food additives [26]. Therefore, the aim to continue this study to analyze real samples and their classification is a current and essential subject that would be very advantageous since spectroscopy is a non-invasive technique requiring small samples. Using deep learning methods would allow the classification of many samples with minimum human assistance, delivering fast results. 

## Figures and Tables

**Table 1:** Molar concentrations for simple solutions.

Numerical Label	Food Additive	Minimum Molar Concentration (M)	Maximum Molar Concentration (M)	Number of Samples
1	Acesulfame Potassium	0.00000976	0.0001875	26
2	Aspartame	0.0000625	0.01	41
3	Sodium Benzoate	0.00000683	0.0005	38
4	Potassium Sorbate	0.000000976	0.000195	32
5	Saccharin	0.0000117	0.00232	56

**Table 2:** Molar concentrations for mixed solutions.

Numerical Label	Additive in Mixture	Maximum Molar Concentration (M)	Minimum Molar Concentration (M)	Number of Samples
1	Acesulfame potassium Aspartame	0.0000116 0.00000488	0.000234 0.000058	15
2	Acesulfame potassium Sodium benzoate	0.0000145 0.0000145	0.00035 0.0000549	31
3	Acesulfame potassium Potassium sorbate	0.0000146 0.00000548	0.000234 0.0000877	26
4	Acesulfame potassium Saccharin	0.00000584 0.00000244	0.000234 0.0000145	21
5	Aspartame Sodium benzoate	0.0000145 0.00058	0.000058 0.000234	12
6	Aspartame Sodium benzoate	0.00000976 0.00000976	0.00375 0.000156	22
7	Aspartame Saccharin	0.00000488 0.00000244	0.000117 0.000058	13
8	Sodium benzoate Potassium sorbate	0.0000244 0.0000122	0.00156 0.0000781	13
9	Sodium benzoate Saccharin	0.00000584 0.00000244	0.000234 0.000058	29
10	Potassium sorbate Saccharin	0.0000072 0.0000145	0.000117 0.000234	29

**Table 3:** The testing accuracy of the neural networks for one-additive solutions.

Neural Network	Mean Testing Accuracy (%)	Standard Deviation (%)
ANN—with 1 hidden layer	97.41	2.04
ANN—with 2 hidden layers	99.67	1.02
CNN—1 convolution layer	99.35	1.36
CNN—2 convolution layers	99.03	1.56
CNN—3 convolution layers	99.67	1.02
CNN—1 convolution and a pooling layer	99.03	1.56
CNN—2 convolution and 2 pooling layers	99.67	1.02
CNN—3 convolution and 3 pooling layers	99.35	2.03

**Table 4:** The mean validation accuracy of the neural networks for one-additive solutions.

Neural Network	Mean Validation Accuracy (%)	Standard Deviation (%)
ANN—with 1 hidden layer	98.06	3.11
ANN—with 2 hidden layers	98.38	1.7
CNN—1 convolution layer	99.67	1.02
CNN—2 convolution layers	99.05	1.52
CNN—3 convolution layers	99.35	1.36
CNN—1 convolution and a pooling layer	98.7	1.66
CNN—2 convolution and 2 pooling layers	99.67	1.02
CNN—3 convolution and 3 pooling layers	99.35	1.36

**Table 5:** The mean testing accuracy of the neural networks for two-additive solutions.

Neural Network	Mean Testing Accuracy (%)	Standard Deviation (%)
ANN—with 1 hidden layer	90.55	5.88
ANN—with 2 hidden layers	88.88	7.04
CNN—1 convolution layer	91.11	4.86
CNN—2 convolution layers	89.44	6.25
CNN—3 convolution layers	89.72	4.90
CNN—1 convolution and a pooling layer	91.94	4.02
CNN—2 convolution and 2 pooling layers	90.83	6.00
CNN—3 convolution and 3 pooling layers	92.22	4.30

**Table 6:** The mean validation accuracy of the neural networks for two-additive solutions.

Neural Network	Mean Validation Accuracy (%)	Standard Deviation (%)
ANN—with 1 hidden layer	88.33	6.52
ANN—with 2 hidden layers	88.05	7.86
CNN—1 convolution layer	92.5	5.56
CNN—2 convolution layers	95.27	2.63
CNN—3 convolution layers	90.52	3
CNN—1 convolution and a pooling layer	94.16	3.05
CNN—2 convolution and 2 pooling layers	93.88	4.3
CNN—3 convolution and 3 pooling layers	92.22	5.82

**Table 7:** The mean testing accuracy of the neural networks for all samples.

Neural Network	Mean Testing Accuracy (%)	Standard Deviation (%)
ANN—with 1 hidden layer	92.83	3.35
ANN—with 2 hidden layers	92.83	2.51
CNN—1 convolution layer	91.79	2.46
CNN—2 convolution layers	90.14	4.78
CNN—3 convolution layers	92.38	1.48
CNN—1 convolution and a pooling layer	89.99	4.10
CNN—2 convolution and 2 pooling layers	91.78	2.46
CNN—3 convolution and 3 pooling layers	91.79	3.93

**Table 8:** The mean validation accuracy of the neural networks for all samples.

Neural Network	Mean Validation Accuracy (%)	Standard Deviation (%)
ANN—with 1 hidden layer	92.83	2.96
ANN—with 2 hidden layers	91.79	4.23
CNN—1 convolution layer	93.88	3.1
CNN—2 convolution layers	93.88	2.16
CNN—3 convolution layers	93.43	2.01
CNN—1 convolution and a pooling layer	89.1	4.92
CNN—2 convolution and 2 pooling layers	93.43	2.01
CNN—3 convolution and 3 pooling layers	91.34	2.61

**Table 9:** Shapiro–Wilk results for one-additive solutions.

Neural Network	*p*-Value (Testing)	*p*-Value (Validation)
ANN—with 1 hidden layer	0.0122682	0.0004833
ANN—with 2 hidden layers	0.0000001	0.0002539
CNN—1 convolution layer	0.0000046	0.0000001
CNN—2 convolution layers	0.0000471	0.0000639
CNN—3 convolution layers	0.0000001	0.0000046
CNN—1 convolution and a pooling layer	0.0000471	0.0001686
CNN—2 convolution and 2 pooling layers	0.0000001	0.0000001
CNN—3 convolution and 3 pooling layers	0.0000001	0.0000046

**Table 10:** Dunn test results for one-additive solutions (testing).

	ANN1	ANN2	CNN1	CNN2	CNN3	CNN1 *	CNN2 *	CNN3 *
ANN1	1	0.042153	0.171482	0.519035	0.042153	0.519035	0.042153	0.056751
ANN2		1	0.999995	0.99959	1	0.99959	1	1
CNN1			1	0.999995	0.999995	0.999995	0.999995	0.999995
CNN2				1	0.99959	1	0.99959	0.99959
CNN3					1	0.99959	1	1
CNN1 *						1	0.99959	0.99959
CNN2 *							1	1
CNN3 *								1

* convolutional layers followed by pooling layers.

**Table 11:** Shapiro–Wilk results for two-additive solutions.

Neural Network	*p*-Value (Testing)	*p*-Value (Validation)
ANN—with 1 hidden layer	0.4429163	0.0513117
ANN—with 2 hidden layers	0.0071524	0.5728397
CNN—1 convolution layer	0.2328355	0.2961086
CNN—2 convolution layers	0.1990748	0.2868703
CNN—3 convolution layers	0.4518404	0.18452
CNN—1 convolution and a pooling layer	0.3295496	0.0189711
CNN—2 convolution and 2 pooling layers	0.0287601	0.1559415
CNN—3 convolution and 3 pooling layers	0.2902561	0.3054604

**Table 12:** Shapiro–Wilk results for all samples.

Neural Network	*p*-Value (Testing)	*p*-Value (Validation)
ANN—with 1 hidden layer	0.2519468	0.270485
ANN—with 2 hidden layers	0.231186	0.0003805
CNN—1 convolution layer	0.4585191	0.1940653
CNN—2 convolution layers	0.16092	0.4851084
CNN—3 convolution layers	0.033043	0.4402599
CNN—1 convolution and a pooling layer	0.0161031	0.8760706
CNN—2 convolution and 2 pooling layers	0.3432861	0.1977461
CNN—3 convolution and 3 pooling layers	0.024538	0.4726183

**Table 13:** One-way ANOVA results (testing dataset).

ANOVA Parameters	Accuracy Dataset for Two-Additive Solutions	Accuracy Dataset for All Samples
*p*-value (testing)	0.8582	0.3733
Degree of freedom (df) within groups	72	72
Degree of freedom (df) between groups	7	7
F ratio	0.4626	1.0986
F critic	2.1397	2.1397

**Table 14:** One-way ANOVA results (validation dataset).

ANOVA Parameters	Accuracy Dataset for Two-Additive Solutions	Accuracy Dataset for All Samples
*p*-value (validation)	0.01526	0.01503
Degree of freedom (df) within groups	72	72
Degree of freedom (df) between groups	7	7
F ratio	2.7	2.7078
F critic	2.1397	2.1397

**Table 15:** Tukey’s HSD test results for two-additive solutions (validation).

	ANN2	ANN1	CNN3	CNN3 *	CNN1	CNN2 *	CNN1 *	CNN2
ANN2	0	0.1702	1.5123	2.5530	2.7232	3.5742	3.7444	4.4252
ANN1		0	1.3421	2.3828	2.553	3.4040	3.5742	4.2550
CNN3			0	1.0406	1.2108	2.0618	2.2320	2.9128
CNN3 *				0	0.1702	1.02120	1.1914	1.8722
CNN1					0	0.8510	1.0212	1.7020
CNN2 *						0	0.1702	0.8510
CNN1 *							0	0.6808
CNN2								0

* convolutional layers followed by pooling layers.

**Table 16:** Tukey’s HSD test results for all samples (validation).

	CNN1 *	CNN3 *	ANN2	ANN1	CNN3	CNN2 *	CNN1	CNN2
CNN1 *	0	2.2346	2.6816	3.7244	4.3203	4.3203	4.7673	4.7673
CNN3 *		0	0.4469	1.4897	2.0857	2.0857	2.5326	2.532
ANN2			0	1.0428	1.6387	1.6388	2.0857	2.0857
ANN1				0	0.5959	0.5959	1.0428	1.0428
CNN3					0	0	0.44693	0.4469
CNN2 *						0	0.44693	0.4469
CNN1							0	0
CNN2								0

* convolutional layers followed by pooling layers.

**Table 17:** Number of possible mixtures and estimated preparation time.

Number of Additives	Possible Mixtures	Minimum Number of Samples/Class	Estimated Time for Sample (Minute)
1	5	25	875
2	10	25	1750
3	10	25	1750
4	5	25	875
5	1	25	175

## Data Availability

Data are available upon request from the corresponding authors.

## Appendix A

Table A1 presents the selected food additives alongside the molecular formula and molar mass. The molar mass is necessary for the calculation of the molar concentration of the samples. It is important to select the volume of the solvent and calculate the mass of the solute that needs to be dissolved into the solvent to obtain a sample with the desired molar concentrations.

**Table A1:** Food additives.

Food Additive	Type of Additive	Molar Mass (g/mol^−1^)	Molecular Formula
Acesulfame Potassium	Sweetener	202.242	C_4_H_4_KNO_4_S
Aspartame	Sweetener	294.3	C_14_H_18_N_2_O_5_
Saccharin	Sweetener	183.18	C_7_H_5_NO_3_S
Potassium Sorbate	Preservative	150.22	C_6_H_7_KO_2_
Sodium Benzoate	Preservative	144.11	C_7_H_5_NaO_2_

Table A2 presents the calculated and measured mass of the solute necessary for obtaining a specific molar concentration for each one of the five additives.

**Table A2:** Mass of solute.

Food Additive	Molar Concentration (M)	Calculated Mass (g)	Measured Mass (g)
Acesulfame Potassium	0.1	0.1006	0.1006
Aspartame	0.01	0.0147	0.0144
Saccharin	0.1	0.0915	0.0916
Potassium Sorbate	0.1	0.0751	0.0752
Sodium Benzoate	0.9	0.0648	0.06483
